# Inflammasome Inhibitors

**DOI:** 10.3390/molecules26226912

**Published:** 2021-11-16

**Authors:** Massimo Bertinaria

**Affiliations:** Department of Drug Science and Technology, University of Turin, Via Giuria 9, 10125 Torino, Italy; massimo.bertinaria@unito.it; Tel.: +39-011-6707146

In recent years, the interplay between the activation of the immune system, the development of chronic inflammation and the onset and progression of many different diseases has been studied extensively [1]. A seminal study by Jürg Tschopp and collaborators describing the formation of a yet unknown intracellular multiprotein complex, named “the inflammasome”, was published in 2002 [2]. The generation of this complex made of NLRP1 (nucleotide-binding oligomerization domain, leucine rich repeat and pyrin domain containing protein-1, also known as NALP-1), ASC (apoptosis-associated speck-like protein containing a caspase-recruiting domain) and procaspase-1 was sufficient to activate the cysteine protease caspase-1 and to trigger the release of interleukin(IL)-1β, a powerful pro-inflammatory cytokine [2]. During the last two decades, several studies have led to the discovery of additional inflammasome complexes that assemble in response to distinct stimuli [3]. Among them, the NLRP3 inflammasome is currently the most widely studied. The dysregulation of the NLRP3 inflammasome signaling was the first to be linked to human pathologies such as the autoinflammatory disease known as Muckle–Wells syndrome and the chronic inflammation found in gout [4,5]. Since then, overexpression or overactivation of this inflammasome was found in many different diseases both at peripheral and central levels. Notably, dysregulation of inflammasome activation can contribute to a multitude of inflammatory diseases, as well as to auto-immune disorders, cardiometabolic diseases, microbiome homeostasis, cancer, neurological disorders, etc.

In this Special Issue, the emerging role played by different inflammasomes in inflammatory bowel disease (IBD) is critically reviewed [6]. Xu et al. perform an analysis of studies focusing on the role of the inflammasomes in the maintenance of gut homeostasis and the development of IBD. Interestingly, in spite of some studies showing a potentially protective role by NLRP3 and NLRP6 inflammasomes in colitis, most of the recent literature converge toward the view that the overactivation of NLRP3 exacerbates intestinal inflammation leading to colitis. Multiple mechanisms control and modulate NLRP3 activity and the consequent release of pro-inflammatory cytokines IL-1β and IL-18. The role played by genetic factors in the onset of Chron’s disease and colitis is efficiently surveyed by the authors. Deficiency of factors such as immunity-related GTPase family M (IRGM), receptor interacting protein kinase1 (RIPK1), Bruton’s tyrosine kinase (BTK), protein tyrosine phosphatase non-receptor type 2 (PTPN2), COMM domain containing protein10 (COMMD10), NADPH oxidase and of micro RNA miR-223 result in increased inflammasome activation in the colon and of associated colonic and systemic inflammation. Furthermore, recent studies demonstrated that gain-of-function mutations (R779C) in NLRP3 or loss-of-function mutation in CARD8 and IL-10R are involved in the onset of IBD and that a key inflammatory role in these pathologies is played by the overproduction of IL-1β. Inhibition of NLRP3 or IL-1β proved beneficial in some of the described conditions, thus evidencing the importance of the NLRP3-IL-1β axis. Other inflammasomes also contribute to intestinal homeostasis and to the control of microbiota composition. The activation of NLRP6, Pyrin, AIM2 and NLRC4 inflammasomes have all been implicated in the pathogenesis of IBD; however, further studies are needed to fully understand their role. The authors discuss the possible application of therapeutic inhibition of inflammasomes, or their downstream cytokines, in treatment of IBD as emerging from data obtained in patients and animal models. Chronic intestinal inflammation and excessive IL-1β release have been also associated with colorectal cancer (CRC) and colitis associated cancer (CAC). A recent study found that the overexpression of NLRP3 is evidenced in CRC-positive tissue in patients; moreover, this expression is correlated with shorter lifespans and poorer prognosis [7], thus highlighting a possible role for NLRP3 in these forms of cancer. The review by Ngui et al. [8] describes the interplay between the aryl hydrocarbon receptor (AhR) and NLRP3 as a novel and promising topic in intestinal inflammation and colon cancer. In their review, the authors describe different signaling pathways through which the activation of AhR inhibits the expression of NLRP3. The therapeutic potential of natural agonists of AhR is also briefly discussed.

The potential of NLRP3 inhibition for the treatment of cardiovascular diseases is a growing field of research which has attracted interest both from academia and industry. The release of damage-associated molecular patterns (DAMPs), also known as alarmins, due to ischemia and postischemic damage promotes the expression and activation of NLRP3 inflammasome. Moreover, in other cardiovascular diseases such as hypertension and atherosclerosis or in chronic diseases associated with an increased risk of developing cardiovascular complications, such as obesity and diabetes, priming of the inflammasome is promoted by metabolites and/or neurohormonal activation. These factors all concur to exacerbate the inflammatory response mediated by NLRP3 activation in the cardiovascular system. In their review, Mezzaroma and colleagues [9] guide the reader through a journey into the role played by the NLRP3 inflammasome and its related cytokines in the heart and in different cardiovascular diseases. Starting from the early observations that the glyburide-derivative 16673-34-0 was able to protect mice from cardiac injury, extensive research was conducted both with new NLRP3 inhibitors as well as with drugs able to modulate different stages of NLRP3 signaling. Promising results were obtained with different molecules and some of them advanced to clinical trials. The efficacy of prototypical inflammasome inhibitors and of interleukin-blocking therapy in both preclinical animal models and in clinical trials are surveyed and extensively discussed.

In 2013, working with an *nlrp3*^−/−^ mouse model, Heneka et al. highlighted the role of the NLRP3 inflammasome in Alzheimer’s disease (AD) [10]. This seminal work opened a new field of research on the relationship between immune system-mediated inflammation and neurodegenerative diseases. Since this discovery, the involvement of the inflammasomes in the onset and progression of different neurodegenerative diseases has been extensively studied. The NLRP3 and the NLRP1 inflammasomes have been both found to contribute to the pathogenesis of AD and the associated neuroinflammation. This response is not only mediated by reactive microglia and astrocytes but it is also sustained by the activation of peripheral immune system. The activation of the NLRP3 inflammasome both through the canonical or non-canonical pathway is also found in patients suffering with multiple sclerosis, leading to the release of inflammatory cytokines. In Parkinson’s disease (PD), a more complex scenario has emerged from different studies. While in animal models, NLRP3 expression and activation appears to be significantly related to the severity of PD, working with PD patients and healthy controls, contrasting results were obtained. In this context, further studies are needed to clarify the contribution of inflammasome to the onset and progression of PD. So far, different studies evidenced an increased neuroinflammation both in patients and in a transgenic murine model of amyotrophic lateral sclerosis (ALS); however, it is not clear whether NLRP3 or other inflammasomes are responsible for this response. In this Special Issue, Piancone et al. give us a concise and updated summary of the research performed to elucidate the role of inflammasomes in neurodegenerative diseases in the last decade [11].

A growing research interest is directed toward the study of the involvement of inflammasomes in hematopoietic homeostasis and hematological diseases such as myelodysplastic syndromes (MDs), myeloproliferative neoplasms (MPNs) and graft-versus-host disease (GVHD). Yang et al. efficiently recapitulate the effects mediated by inflammasomes on the hematopoietic system [12]. In their paper, the role of NLRP1, AIM2, NLRP3 and NLRP12 inflammasomes activation or inhibition in hematopoietic stem/progenitor cells (HSPCs) is extensively reviewed. The authors describe how the activation of different inflammasomes in HSPCs maintenance and differentiation is implicated in the pathogenesis of neutrophilia and anemia during chronic inflammatory diseases, suggesting a pharmacological target for therapeutic intervention. Moreover, aberrant NLRP3 activation, typical of HSCs during physiological aging, can be a new target for NLRP3 inhibitors aimed at increasing the maintenance and the regenerative capacity of aged HSCs. 

The activation of different inflammasomes is tightly regulated at the physiological level. Recent reviews describe the current knowledge on the subject which, for most of the inflammasomes, is still to be fully understood and elucidated [13]. The NLRP3 inflammasome is the most studied and much progress has been made in the knowledge about its regulatory mechanism [14]. One of the main points that remains to be elucidated is the role of ATP-binding and hydrolysis in inflammasome activation. Sandall and colleagues illustrate the state of the art in this field [15]. In their work, the authors provide a very complete picture on NLR proteins and, more specifically, on the NLRP-subfamily and their dependency on ATP binding for activation, assembling and downstream signaling. The ATP-dependency for assembly and activation is discussed based on recent biochemical and structural studies. The role of key functional motifs in the NACHT domain of the NLR protein for ATP-binding and hydrolysis is carefully examined together with the structural basis and the molecular determinants for inflammasome assembly. Finally, a survey of NLRP3 inhibitors able to act as NLRP3 ATPase inhibitors is reported.

One bottleneck in the discovery of new NLRP3 inhibitors is represented by the complex mechanism of activation which entails “canonical”, “non-canonical” and “alternative” activation. This complexity can hamper the efficient development of selective inflammasome inhibitors complicating the study of their biological activity and the elucidation of their mechanism of action. A much-needed review by Angosto et al. collects and critically discusses the techniques used to study small molecules inhibitors of the inflammasome [16]. The paper focuses both on techniques that can be used for the screening of relatively large series of newly designed molecules and on techniques useful to investigate the mechanism of action of selected compounds. To elaborate on the most studied inflammasome, the ideal NLRP3 inhibitor should be able to bind to NLRP3 protein and impair its oligomerization. As clearly explained in this review, different experiments should be performed in order to ascertain the specificity and the selectivity of compounds acting as NLRP3 inhibitors. The suggested approach consists of an initial screening of protein expression followed by tests aimed at proving the ability of compounds to inhibit cytokine release and cell pyroptosis. A second level of testing should then exclude other possible target in the signaling pathway (e.g., caspases and NF-kB pathway). Finally, as a possible third step, specific measurement of sensor protein binding could be performed via different biophysical or biochemical techniques as described in the article. 

Progress has been made since the publication of the first review on inflammasome inhibitors by Freeman et al. [17]; in this issue, El-Sharkawy et al. provide us with an update on inflammasome inhibition by small molecules [18]. In this paper, focused on selective NLRP3 inhibitors, both covalent and non-covalent inhibitors are presented and their mechanism of action is briefly discussed. The discovery of MCC950 (also known as CRID3 and CP-456773) as a potent and selective NLRP3 inhibitor started a great research effort toward the synthesis of MCC950 analogues with some MCC950-derived molecular probes designed for the elucidation of its binding to NLRP3. Recent research related to the discovery of new NLRP3 inhibitors, beyond MCC950, is surveyed too.

Many compounds able to block NLRP3 signaling discovered so far come from natural products research [19,20]. In this Special Issue, some research articles focused on the study of natural compounds acting as NLRP3 inhibitors are included. Tomani et al. studied the anti-inflammatory effects of *Hibiscus noldae* Baker f., a component of traditional preparations used in Rwanda to treat several inflammatory conditions [21]. Using a bio-guided fractionation approach, the authors reveal the ability of *H. noldae* extracts to inhibit IL-1β, IL-6 and pyroptosis in vitro and to reduce the expression of pro-caspase-1, a key protein in the NLRP3 signaling pathway. Results suggest that several polyphenolic components, including caffeic acid and isoquercetin, may be responsible for these effects. Choi et al. investigate the mechanism of action of loganin, an iridoid glycoside, known for its anti-inflammatory activity [22]. The authors demonstrate that loganin inhibits NLRP3-dependent IL-1β release triggered by monosodium urate crystals with no effect on TNF and IL-6 levels in vitro. Moreover, when administered orally at 5 and 30 mg/kg, loganin is also able to reduce the release of inflammatory cytokines in two mouse models of gout, showing promising potential for the development of a new treatment for gout. Chalcones are a class of plant secondary metabolites well known for a wide range of biological activities. Their structure contains an α,β-unsaturated carbonyl moiety which entails a typical Michael-acceptor behaviour. Michael acceptors are electrophiles able to act as covalent NLRP3 inhibitors [23,24]; in their study, Leu et al. explore the structure–activity relationship of chalcones as NLRP3 inflammasome inhibitors [25]. After a screening for caspase-1 activation, the authors identify a compound (11Cha1) with a low micromolar IC_50_ which is also able to reduce the release of IL-1β, IL-18 and pyroptotic cell death with a similar potency. Like most of the electrophilic compounds, 11Cha1 is not selective toward NLRP3, showing an inhibition of the priming step as evidenced by its repressive effect on the NF-kB pathway. Further validation of this compound in an in vivo disease model is needed to verify the pharmacological potential of this chalcone. On the basis of the previously discovered immunomodulatory and anti-inflammatory activities of the endogenous antioxidant α-lipoic acid (ALA) and taking into account that chronic inflammation can play an essential role in endometriosis, Di Nicuolo et al. designed a study to look at the effects of ALA on ER-β/NLRP3-mediated signaling in vitro employing two endometriotic cell lines [26]. This is a relatively new potential application for NLRP3-modulating compounds. In this study, the authors demonstrated that ALA significantly reduces ER-β and NLRP3 expression and inhibits NLRP3-mediated secretion of pro-inflammatory interleukins. Moreover, the authors found an interesting effect of ALA on the expression of the adhesion molecule ICAM-1 and matrix-metalloproteases MMP2 and MMP9, which are produced in response to IL-1β secretion and promote cell invasiveness.

In an effort to develop new chemical templates endowed with NLRP3 selectivity, Gastaldi et al. synthesised a library of benzo[d]imidazole-2-one compounds designed by pharmacochemical hybridization [27]. The authors merged the structure of the previously discovered NLRP3 ligands INF39 and HS203873 to obtain non-covalent NLRP3 inhibitors. The biological screening allowed the identification of four promising hit compounds able to concentration-dependently inhibit NLRP3-dependent pyroptotic cell death in human macrophages. These compounds were also able to partially block the ATPase activity of NLRP3. Computational simulations were applied for building a complete model of the NLRP3 inactive state and for identifying possible binding sites available to the tested compounds. A putative binding pocket was identified and a binding mode, which could be helpful for the design of improved NLRP3 blockers, was proposed.

Dapansutrile (also known as OLT1177^®^) is one NLRP3 inhibitors now in clinical trial for the treatment of systemic inflammation and knee osteoarthritis. In this issue, Aliaga and colleagues present the results obtained by studying OLT1177 in a mouse model of severe ischemic cardiomyopathy [28]. This study showed how chronic treatment with the NLRP3 inhibitor can prevent left ventricular diastolic dysfunction and preserved β-adrenergic responsiveness. According to the authors, the obtained results suggest a possible application of this inhibitor in patients with heart failure.

Finally, Das et al. present and discuss a collection of papers describing the effect of either known drugs or experimental compounds which have been tested in NLRP3-driven disease models in vivo [29].

Given the fundamental importance of NLRP3 signaling in several diseases, the main focus today is on the development of NLRP3 inhibitors. Inhibitors of the inflammasome are being developed by both industry and academia with some NLRP3 inhibitors currently in clinical trials. The outcome of these trials and the disclosure of new chemical models will further drive the search for new NLRP3 blockers in the coming years.

## Data Availability

Not applicable.
